# Strong interfacial Dzyaloshinskii–Moriya induced in Co due to contact with NiO

**DOI:** 10.1038/s41598-022-16997-4

**Published:** 2022-07-26

**Authors:** M. Kowacz, P. Mazalski, I. Sveklo, M. Matczak, B. Anastaziak, U. Guzowska, A. K. Dhiman, E. Madej, A. Maziewski, P. Kuświk, R. Gieniusz

**Affiliations:** 1 Institute of Molecular Physics Polish Academy of Sciences, Poznań, Poland; 2 Faculty of Physics, University of Bialystok, Białystok, Poland; 3 Jerzy Haber Institute of Catalysis and Surface Chemistry, Polish Academy of Sciences, Kraków, Poland; 4 NanoBioMedical Centre, Adam Mickiewicz University in Poznan, Poznań, Poland

**Keywords:** Ferromagnetism, Magnetic properties and materials, Surfaces, interfaces and thin films

## Abstract

The magnetic properties of NiO/Co/Pt as a function of Co layer thickness were investigated by polar magneto-optical Kerr effect (PMOKE) (magnetometry and microscopy) and Brillouin Light Scattering (BLS) spectroscopy. PMOKE measurements revealed strong surface anisotropy (1.8 mJ/m^2^) favoring perpendicular magnetic anisotropy and asymmetric domain wall propagation explained by anticlockwise chirality. BLS measurements show that this chirality is induced by strong interfacial Dzyaloshinskii–Moriya interaction (+ 2.0 pJ/m). This is one of the highest values reported so far for Co layers surrounded by different layers. The observed chirality is opposite to what has been found in Co/oxide interfaces. These results and data published earlier, indicate that the strength of interfacial Dzyaloshinskii–Moriya interaction increases with the amount of stoichiometric NiO. Therefore, this work shows that NiO is the source of the interfacial Dzyaloshinskii–Moriya interaction.

## Introduction

Currently, the interfacial Dzyaloshinskii–Moriya interaction (iDMI) is intensively studied in magnetic thin films. It is induced by inversion symmetry breaking and stabilizes chiral structures, including skyrmions. The investigation of iDMI in antiferromagnetic (AFM)/ferromagnetic (FM) systems with exchange bias (EB) coupling is of great importance because skyrmions can be stabilized at room temperature (RT) and zero magnetic field in FM films with perpendicular magnetic anisotropy (PMA) by interactions with AFM layers^1^. Moreover, since skyrmions can be created and destroyed with electric fields, insulating materials including oxides are needed as well^2^. The modification of electronic states around Fermi level at the interface between oxide and FM materials and its influence on magnetic properties are nontrivial, so different combinations of layer materials are studied both experimentally and numerically by first principles calculations^3–7^. Antiferromagnetic oxides (AFO) belongs to this group and can be ideal candidates towards the fulfillment of these requirements. However, only a few works so far have paid attention on how the AFO influences the iDMI in ferromagnets. Therefore we will focus on NiO, which strongly pins ferromagnetic spins by exchange bias coupling^8^ and in stoichiometric form is an insulator (note that even single-crystal NiO may deviate from the proper stoichiometry Ni:O ratio (1:1) because of lattice defects (vacancies/interstitials)^9,10^).

Spin textures in exchange biased Au/Co/NiO systems show clockwise (CW) chirality; as inferred using asymmetric domain propagation^11^ and magnetization reversal of micrometric-sized triangles^12^. In those systems, it was expected that the Au layer would not be the main source of interfacial DMI and the chirality would be supported only at the Co–CoO–NiO interface. Taking this into account the strength of iDMI (*D*_*S*_) measured was not so high (*D*_S_ = − 1.1 pJ/m) and could also be affected by the Co oxide layer that appeared at the interface between Co and NiO. On the other hand, the iDMI and EB coupling have also been investigated in Pt/Co/Ni_x_O_y_^13^, where the concentration of the NiO phase was controlled by changing the Ar pressure during deposition. In that case, the maximum of *D*_S_ = − 1.25 pJ/m was reached for 40% concentration of NiO in layers deposited by sputtering Ni_x_O_y_. Therefore, it is important to determine two facts about the nickel oxide layer obtained during pulsed laser deposition (PLD): (1) whether the concentration of stoichiometric NiO on it enhances iDMI; and, (2) which type of chirality does it support.

To investigate the iDMI, we used Brillouin light scattering (BLS) spectroscopy and the asymmetric domain propagation method based on polar magneto-optical Kerr effect (PMOKE) microscopy applying magnetic fields with in-plane and perpendicular components to the sample plane. We demonstrate that NiO(bottom)/Co/Pt(top) shows anticlockwise (ACW) chirality, which confirms earlier assumptions that the NiO layer is a strong source of iDMI and favors opposite chirality (in contrast to other non-antiferromagnetic oxides such as MgO and Al_2_O_3_). The investigation was performed on wedged samples, which enable measurements of iDMI in a wide range of Co thicknesses (*d*_Co_). We found a large iDMI (*D*s =  + 2.0 pJ/m), among the strongest reported on Co layers surrounded with different materials. Moreover, due to strong surface anisotropy contributions at the NiO/Co and Co/Pt interfaces (1.8 mJ/m^2^), PMA holds up to *d*_Co_ ≅ 1.5 nm.

## Results

### Magnetooptical studies

**Figure 1:**
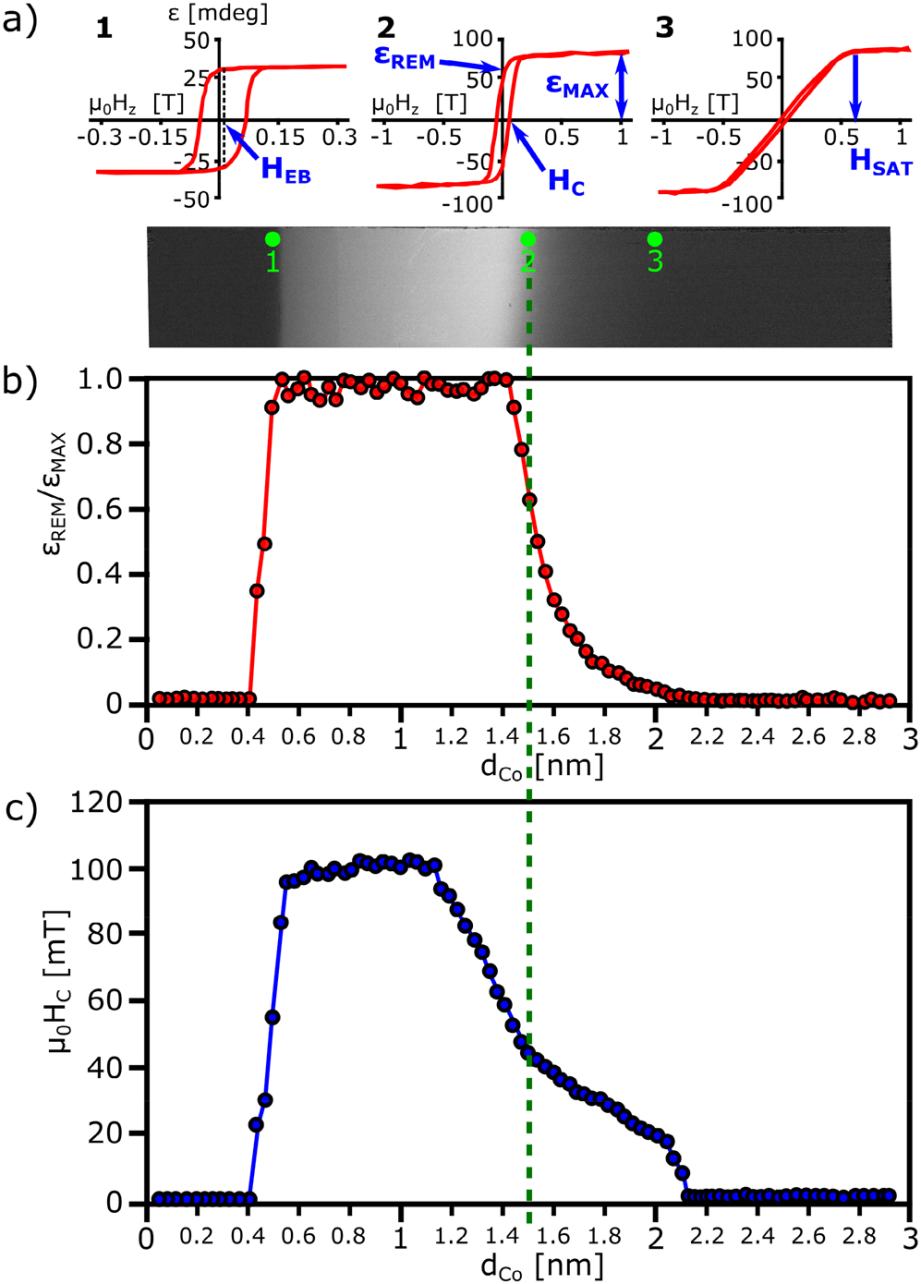
Results of PMOKE measurements. (**a**) Differential PMOKE microscope images registered in remanence after saturation with an out-of-plane magnetic field, where the bright area corresponds to the part of Co layer with PMA. This panel additionally shows the exemplary PMOKE hysteresis loops measured for *d*_*Co*_ = 0.5, 1.5, 2 nm (green dots 1 to 3, respectively). On that loops the definitions of characteristic parameters: remanence (*ɛ*_*REM*_), ellipticity in maximal magnetic field (*ɛ*_MAX_) and saturation field (*H*_SAT_) and exchange bias field (*H*_*EB*_) are given graphically; (**b**,**c**) *d*_*Co*_ dependencies of normalized Kerr signal *ɛ*_REM_/*ɛ*_MAX_ and, coercivity (*H*_*C*_), respectively. The green dashed vertical line indicates the Co thickness for spin reorientation transition (see Fig. 2d).

The magnetic ordering of the Co layer is determined from large field of view PMOKE microscope images (differential with respect to saturated state) registered in remanence after out-of-plane magnetic field saturation (Fig. 1a). The bright areas in this PMOKE image roughly correspond to the out-of-plane magnetization state; the dark areas, to the in-plane magnetization state of the thick Co layer, and to the non-ferromagnetic state of the thin Co layer. These data show that the PMA is observed in a wide *d*_Co_ range if Co is surrounded by NiO and Pt layers. To analyze this effect, we measured magnetization reversal along the Co wedge (as a function of *d*_Co_) using PMOKE magnetometry. Figure 1a presents three exemplary hysteresis loops for *d*_Co_ = 0.5, 1.5, 2 nm. From these measurements, we were able to determine *d*_*Co*_ dependence of basic magnetic and magnetooptical parameters like coercive field *H*_C_; ellipticity in remanence (*ɛ*_REM_); and, at the maximum magnetic field (*ɛ*_MAX_), saturation field (*H*_SAT_) and exchange bias field (*H*_EB_). These data confirm that the PMA is preserved in a wide *d*_Co_ range showing the rectangular shape of the hysteresis loop *(ɛ*_REM_*/ɛ*_MAX_ = 1) up to the thickness for spin reorientation transition (SRT), *d*_SRT_ ≅ 1.5 nm (Fig. 1b). Above *d*_SRT_, the out-of-plane easy axis direction rotates to easy-plane. Since the deposition process was performed in an external out-of-plane magnetic field, the *H*_EB_ is detected with maximal value for *d*_Co_ ~ 0.5 nm and decreases for smaller Co thicknesses (not shown here). The presence of the EB coupling is also manifested by the large value of *H*_C_, which is much higher than reported for non-oxide HM/Co/HM systems^14^ (Fig. 1c). The reduction of *H*_*C*_ starts at lower *d*_*Co*_ than normalized ellipticity *ɛ*_*REM*_*/ɛ*_*MAX*_ due to strong influence of *d*_*Co*_ on domain propagation field near SRT^8^. While decreasing *d*_Co_ from SRT the magnetization reversal process is realized by domain nucleation mechanism—coercivity wall with large number of nucleation centers, then by domain wall propagation mechanism dominates (see Supplementary materials Fig. S1).

**Figure 2:**
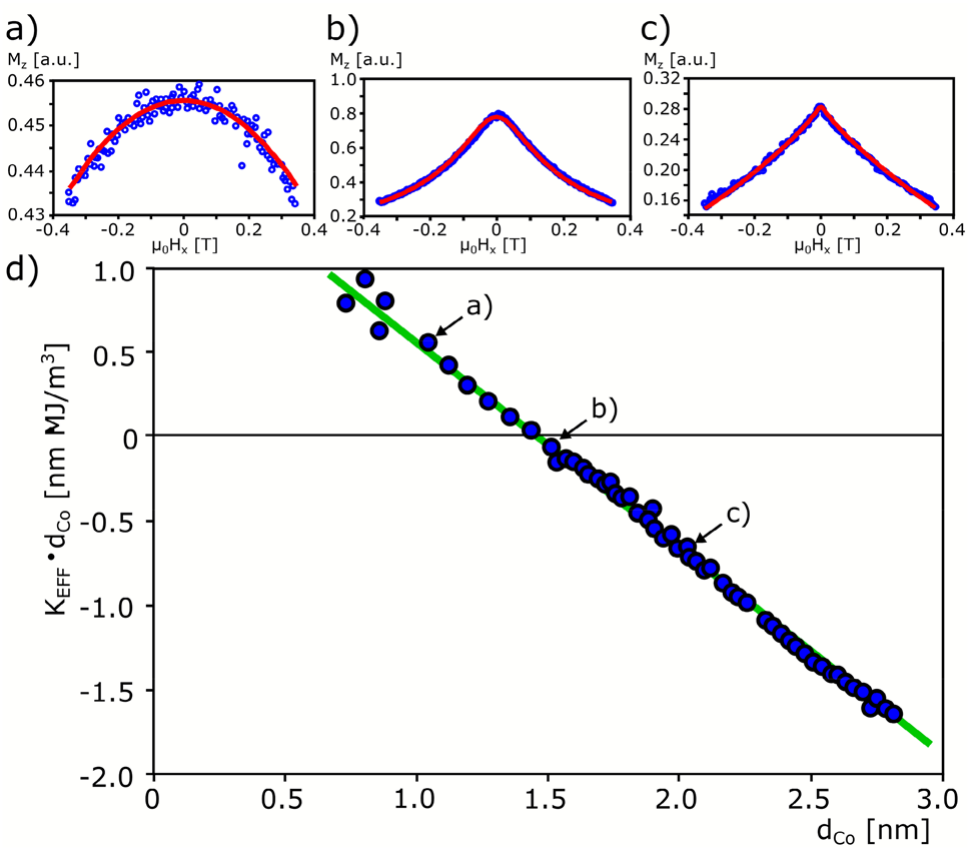
Magnetic anisotropy analysis: (**a**–**c**) exemplary PMOKE signal dependencies as function of in-plane field under a constant 0.15 T out-of-plane field. Red lines—fitting to the Stoner-Wohlfarth model, for selected *d*_Co_ = 1, 1.5, 2 nm, respectively. (**d**) *K*_1EFF_*d*_Co_ vs *d*_Co_. Green line—A linear fit to *K*_1EFF_*d*_Co_ = *K*_V_ *d*_Co_ + *2K*_S_ provides surface (*2K*_S_) and volume (*K*_V_) magnetic anisotropies. Points (**a**–**c**) in panel (**d**) correspond to *d*_Co_ in panels (**a**–**c**), respectively.

For the case of in-plane magnetization, the effective magnetic anisotropy field, *H*_1EEF_, was determined as the saturation field *H*_SAT_ obtained from PMOKE magnetization reversal (see PMOKE loop 3 in Fig. 1a). The magnetic anisotropy constant *K*_*1EFF*_ was calculated according to the relation *K*_1EFF_ = *1/2μ*_0_*H*_1EFF_*M*_S_, where *M*_S_ is the saturation magnetization for bulk cobalt *μ*_0_*M*_s_ = 1.45 T. In the case of out-of-plane magnetization and above *d*_SRT_, where a weak in-plane magnetic anisotropy appears (*d*_Co_ ≤ 2 nm), *H*_1EFF_ was determined by fitting the Stoner–Wohlfarth model^15^ to the PMOKE signal measured as a function of the in-plane magnetic field under a constant out-of-plane magnetic field to ensure single domain state (see Fig. 2a–c).

Figure 2d shows the *d*_Co_ dependence of *K*_1EFF_ *d*_Co_. We found that for thin Co layers (*d*_Co_ ~ 0.6 nm) the *K*_1EFF_ is much stronger than for other systems (e.g. Pt/Co/Pt, Pt/Co/Ir, Ir/Co/Pt) ^14,16^. This strong PMA is associated with the presence of NiO and Pt as buffer and cover layers, respectively. The Pt layer supports the PMA owing to large spin–orbit coupling^17^; at the same time, hybridization between Co and O atoms at the NiO/Co interface can also be a source of PMA^18^. However, it was recently demonstrated that the AFM layer in the case of Co/NiO bilayer gives an additional contribution to magnetic anisotropy^19^, which vanishes above Néel temperature of CoO/NiO layers^20^. Therefore, this coupling has a sufficiently strong surface contribution, which leads to the Co layer being magnetized perpendicularly to the sample plane in wider *d*_Co_ ranges than in other polycrystalline HM/Co/HM systems^19^. To determine the surface (2*K*_S_) and volume (*K*_V_) anisotropy contributions we used a linear fit to the *K*_1EFF_*d*_Co_(*d*_Co_) dependence (Fig. 2d). From this fitting, we obtained 2*K*_S_ = 1.82 ± 0.06 mJ/m^2^ and *K*_V_ = − 1.23 ± 0.04 MJ/m^3^. Note that *K*_V_ includes the shape anisotropy contribution too. This high 2*K*_S_ value confirms that the surface contribution is a main source of PMA in NiO/Co/Pt. It should be emphasized that this value is also much higher than for similar NiO/Co/Au trilayers systems^11^, Au/Co/NiO^19^, Pt/Co/AlO_x_^21^, Ir/Co/AlO_x_^22^.

**Figure 3:**
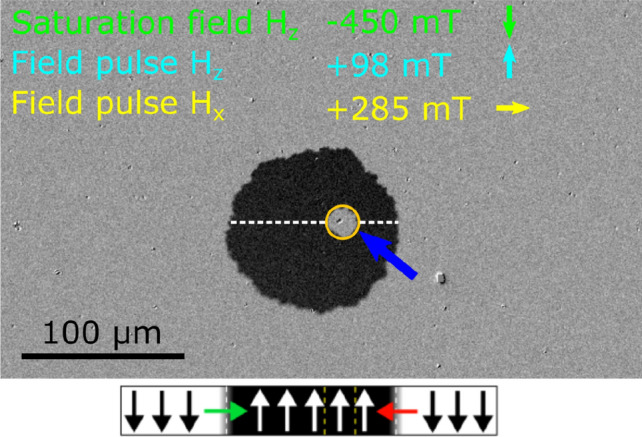
PMOKE difference image of the bubble domain growth with applied in-plane *H*_x_ and out-of-plane *H*_z_ fields for the buffer/NiO(10 nm)/Co(0.9 nm)/Pt(5 nm) system. The values and directions of applied fields are shown in the image. The yellow ring indicates the initial position of the initial bubble domain. Place below the image there is a scheme of the lateral magnetization profile along the dashed white line shown above. The N-DWs are marked by red and green arrows indicating anticlockwise chirality.

Recent results show that NiO in contact with the Co layer might be the source of iDMI^6^; therefore, we measured the domain propagation under a combination of in-plane *H*_x_ and out-of-plane *H*_z_ field pulses. iDMI has been confirmed as an origin of asymmetric domain wall propagation^23^. This effect occurs because the external in-plane magnetic field induces asymmetrical changes of the energy of Néel domain walls (N-DWs)^24^. Therefore, with the right combination of *H*_z_ and *H*_x_, domain walls with core magnetizations pointing along and against *H*_*x*_ move at different speeds causing asymmetric growth of bubble domains. The studies were performed for *d*_Co_ = 0.9 nm where sufficiently large bubble-like domains appear. Because of high coercivity and magnetic anisotropy, it was necessary to use a strong *H*_*z*_ pulse for this experiment. In this experiment, we found such asymmetric domain growth in our samples (Fig. 3) confirming the presence of a strong iDMI. By analyzing the asymmetry of domain wall propagation for different directions of *H*_x_ and *H*_z_ fields, we determined the chirality of NiO/Co/Pt. Our data reveals that spin configuration in the N-DW has ACW chirality, which is related to a positive iDMI constant (*D*_S_ > 0). Note that *D*_S_ sign agrees with data presented in Ref.^13^, which uses the reverse order of surrounding layers, namely Pt(bottom)/Co/Ni_x_O_y_(top), and found negative values of iDMI. For this system, it has also been shown that the magnitude of |*D*_S_| increases with larger concentrations of stoichiometric NiO in the nickel oxide layer. However, use of the sputtering technique for nickel oxide in Ar + O_2_ atmosphere presented in Ref.^13^ did not allow to obtain NiO concentrations larger than 40%. Despite this, a quite strong iDMI with a maximal value of *D*_S_≈-1.2 pJ/m was found. Based on those results, we expected that more stoichiometric NiO would further enhance the iDMI. Therefore, we grew NiO layer using PLD techniques in O_2_ atmosphere reusing earlier parameters^11^. This allows us to check the influence of NiO on the iDMI strength with BLS measurements.

### Brillouin light scattering studies

**Figure 4:**
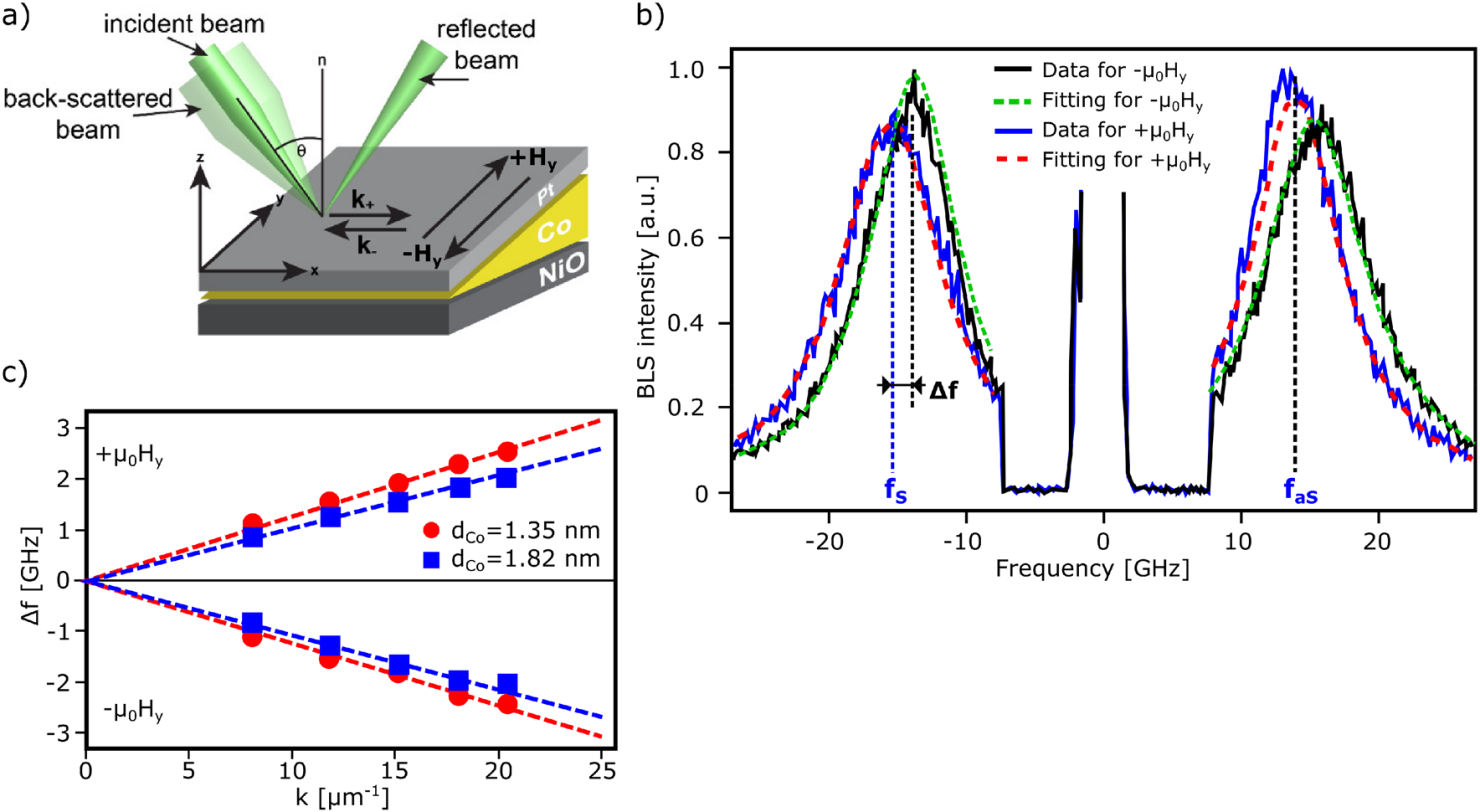
Results of BLS measurements. (**a**) BLS schematic for Damon-Eshbach configuration: magnetic field is applied in the sample plane along y; xz—plane of incidence, *θ*—incidence angle; studied SW wave vector *k* is along x; (**b**) BLS spectrum acquired for *k* = 11.81 µm^−1^, *µ*_0_*H*_y_ = − 0.4 T (black curve) and *µ*_0_*H*_y_ =  + 0.4 T (blue curve) for *d*_Co_ = 1.35 nm with the definition of the characteristic parameters: the Stokes frequency *f*_S_, the anti-Stokes frequency *f*_aS_, frequency difference *Δf*; the dashed red (green) lines represent the peaks fittings to the positive (negative) in-plane field. (**c**) *Δf*(k) dependence measured for positive (+ *µ*_0_H_y_) and negative (*− µ*_0_H_y_) in-plane fields for *d*_Co_ = 1.35 nm (red dots) and 1.82 nm (blue squares); red (blue) dashed lines—linear fits passing through (0;0) for *d*_Co_ = 1.35 nm (1.82 nm). The error of the *Δf*(k) is 0.05 GHz and 0.07 GHz for *d*_*Co*_ = 1.35 and 1.82 nm, respectively.

BLS measurements were performed in the Damon–Eshbach (DE) configuration (see a schematic of the measurement setup in Fig. 4a). Figure 4b shows exemplary BLS spectra for Co layer thicknesses, *d*_Co_ = 1.35 nm. The frequency difference between BLS peaks was measured for two opposite directions of the applied in-plane magnetic field. By comparing curves for opposite field orientation, the difference *Δf* between frequency of Stokes (*f*_S_) and anti-Stokes (*f*_aS_) peaks can be obtained (*Δf* = *f*_S_ – *f*_aS_)(see Fig. 4b).

These measurements were performed for different *k* values by changing the incident angle *ϴ* to determine the effective iDMI constant (*D*_eff_≡*D*_S_/*d*_Co_) from Δ*f*(*k*) relation^25–27^:

1 $$ {\Delta}f\left( k \right) = {2}\gamma D_{{{\text{eff}}}} k/{\pi}M_{{\text{S}}} $$

where *γ* is the gyromagnetic ratio, *M*_s_ magnetization saturation of magnetic layer (for Co we used μ_0_*M*_S_ = 1.45 T, *γ* = 170 GHz/T). Figure 4c shows examples of Δ*f*(*k*) dependencies for different Co thicknesses (*d*_Co_ = 1.35 and 1.82 nm) measured for positive and negative directions of the applied in-plane magnetic fields. From the analysis of the results shown in Fig. 4c we found this typical behavior for samples with iDMI: (i) the sign of the slope in *Δf(k)* dependence reverses with the field direction; (ii) this slope (corresponds to the increase of *D*_eff_) increases as *d*_Co_ decreases. Since the BLS measurements were performed along the Co wedge, the value of *D*_eff_ was determined for a wide *d*_Co_ range.

**Figure 5:**
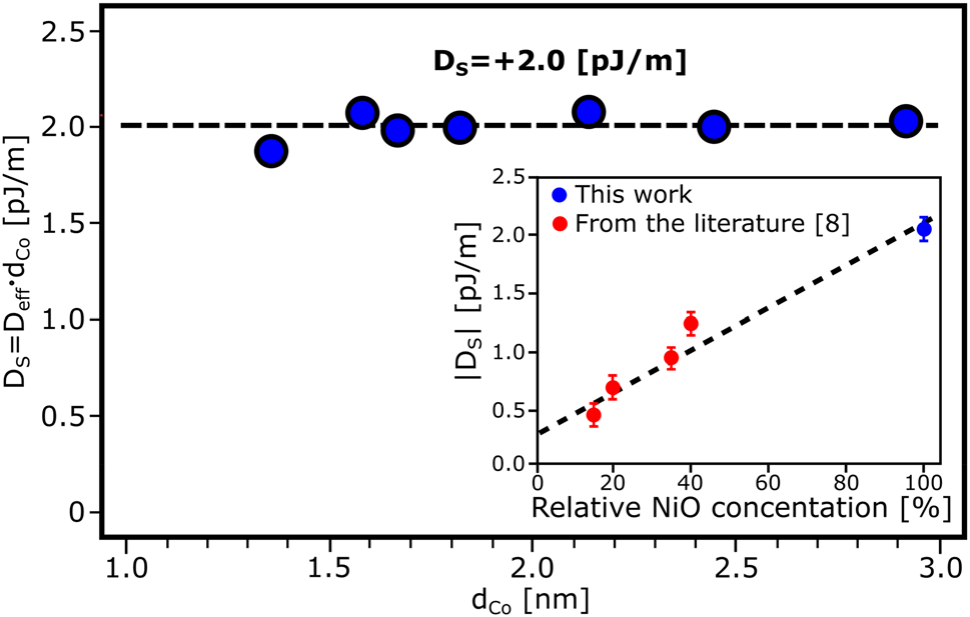
Magnitude of iDMI (*D*_S_) as a function of cobalt thickness (*d*_Co_) for NiO/Co/Pt. The inset shows *D*_s_ as a function of the relative concentration of NiO phase; for Pt/Co/Ni_x_O_y_ (data are taken from^13^, red filled circle, negative *D*s) and for NiO/Co/Pt (this work, blue filled circle positive *D*s) samples, respectively. The dashed line is a linear fit.

Figure 5 presents the dependence of the product *D*_eff_*⋅d*_Co_ as a function of *d*_Co_. The iDMI constant (*D*_s_) can be determined since this value is independent of *d*_Co_ in the measured range. The value obtained, *D*_S_ =  + 2.0 pJ/m, is higher than those observed in similar systems (e.g. Au/Co/NiO trilayers where *D*_S_ = − 1.11 pJ/m^8,12^ and Pt/Co/Ni_y_O_x_ where − 0.5 < *D*_S_ < − 1.2 pJ/m depending on Ar pressure during deposition^13^). Note that, as the order of the cobalt layer covers is reversed^13^, the sign of *D*_S_ changes too; therefore, the chirality set by NiO is the same. Moreover, considering the data from Ref.^13^ and our data, we infer that the magnitude of the *D*_*S*_ increases together with the concentration of NiO phase. It should be mentioned that the relative NiO concentration in^13^ was obtained as a ratio of 529.9 eV to 532.6 eV XPS peaks of O 1 s spectra, whereas in^11^ 100% NiO concentration was deduced from Ni 2p spectra. The composition of NiO layer deposited by PLD was also evaluated from the reference sample (50 nm of NiO) described in the Supplementary Materials (Fig. S2). This allows us to to show that there is linear dependence of *D*_S_ on stoichiometric NiO phase concentration (inset in Fig. 5). Note that the deposition process of NiO was much different for both systems (Ni_x_O_y_ was deposited by reactive sputtering^13^, while NiO was deposited by PLD^11^); therefore correlation between iDMI and the amount of NiO phase might be a universal relationship. This reveals that DMI can be tailored by tuning the quality of the NiO layer with respect to defects and/or the presence of Ni_2_O_3_ phase in the nickel oxide layer. Interestingly, the NiO phase is responsible for stronger iDMI favoring clockwise chirality if it is on the Co layer and anticlockwise chirality when Co is on top of the NiO layer. This might be quite surprising because it is the opposite of what was previously found for different systems (oxide/Co/Pt^21^ and Pt/Co/oxide^21,28–30^). Considering that the iDMI originates from an exchange interaction between two neighboring FM spins coupled via a neighboring layer, we suppose that the additional contribution to iDMI comes from the interaction between Co and the antiferromagnetic NiO phase.

## Conclusion

We have investigated the changes of the magnetic properties in the NiO/Co/Pt trilayer as a function of Co layer thickness. Magnetooptical studies reveal significant value of the magnetic anisotropy occurring in the PMA region and anticlockwise spin chirality within the Néel domain walls. BLS measurements confirm the presence of iDMI with a high value of *D*_S_ =  + 2.0 pJ/m. This value is higher than that of similar systems where the Co layer was in contact with NiO and systems where Co was in contact with other nonmagnetic oxides. The dependence between iDMI and the relative phase concentration of NIO shows that NiO is an additional source of DMI with a chirality that is opposite to that of other nonmagnetic oxides.

## Methods

Samples with nominal structures Ti(4 nm)/Au(60 nm)/NiO(10 nm)/Co(0 ÷ 3 nm, wedge)/Pt(5 nm) were deposited on thermally oxidized silicon substrate using magnetron sputtering (Ti, Au, Pt and Co layers) or PLD (NiO layer) in a UHV multichamber system with base pressure below 2 × 10^−8^ mbar. The nominal thicknesses of the Ti, Au, Co, Pt, and NiO were calculated using deposition time and the deposition rate determined by X-ray reflectivity and/or profilometer measurements from calibration samples. The Co thickness was linearly varied along the sample with a slope of 0.15 nm/mm. This was realized using a moving shutter (slightly above the substrate) with constant velocity during deposition. For correct stoichiometry, the NiO layer was deposited in an oxygen-rich atmosphere (*p*_O_ = 1.5 × 10^−5^ mbar) in a separate UHV chamber^11^. After NiO deposition, the sample was transferred to magnetron sputtering chamber without breaking UHV conditions (during transfer, *p* ≤ 5 × 10^−8^ mbar). This reduces oxidation of the Co layer, as discussed in our earlier work^8^. During deposition, the sample was kept in an external out-of-plane magnetic field *μ*_0_*H*_dep_ = − 0.19 T to increase EB coupling between Co layer and NiO layers. As the NiO layer is covered by the Co and Pt layers the evaluation of the NiO stoichiometry is not possible from direct XPS measurements. To verify the composition of the NiO layer the XPS measurements were performed on the 50 nm thick NiO deposited onto Si substrate in the same condition as for the full layer stack. The results are presented in the Supplementary Materials (Fig. S2).

PMOKE magnetometry measurements were performed using a laser beam with wavelength 635 nm focused on 0.3 mm spot at nearly normal light incidence.

A PMOKE microscope equipped with a CCD camera and an appropriate digital image processing software (in PMOKE configuration the measured signal intensity is proportional to the out-of-plane magnetization component) were used to: (i) record large field of view image of remanent magnetization distributions after out-of-plane saturation; (ii) determine local magnetization loops in the whole sample area with 10 µm lateral resolution. Asymmetric domain propagation was measured using PMOKE microscope with a pulsed magnetic field applied with simultaneous out-of-plane and in-plane components. All recorded PMOKE images were presented as differential images that were obtained after, pixel-by-pixel, gray scale intensity subtraction of the reference image obtained in initial state (with small bubble domain).

BLS experiments were performed in the backscattering configuration, illuminating the sample with p-polarized laser beam with a wavelength *λ* = 532 nm and beam spot size about 20 μm at variable incident angle *Θ*. In this geometry, the value of excited (absorbed) in-plane magnon wave vector *k* is equal to 4π/*λ*sin*Θ*. The laser power was less than 70 mW to avoid sample overheating. The scattered light was collected by a focusing lens and its spectrum was analyzed by a Sandercock-type multi-pass tandem Fabry–Perot TFP-2 HC interferometer. The external magnetic field *H*_y_ was applied in the sample plane and normal to the scattering plane of the laser light. More detailed description of used BLS measurements is given in^31^.

All measurements were performed at RT.

## Supplementary Information

Supplementary Information.

## Data Availability

All results presented in this paper are available from the corresponding author on reasonable request.
