# Supplementary material for: Strong interfacial Dzyaloshinskii–Moriya induced in Co due to contact with NiO
Source: Sci Rep. 2022 Jul 26;12:12741. doi: 10.1038/s41598-022-16997-4 (PMC9325689; doi:10.1038/s41598-022-16997-4)
Supplement: Supplementary file 1 — Supplementary Information. [file 41598_2022_16997_MOESM1_ESM.doc]

**Supplementary material**

***S1. Magnetization reversal process***

Remanence domain structures are presented in Fig.S1. Domain imaging during magnetization reversal driven by *H*_z_<0 field pulses (preferring black domain) applied to the sample initially saturated by *H*_z_>0 (white domain state) is illustrated in Fig.S1. Fig.S1a shows Co wedge remanence image similar as in Fig.1 in the main text. The images in figure S1b were registered from the region (about 2.8x2 mm^2^) of the Co wedge corresponding to Co thickness 1.0 < *d*_Co_ < 1.4 nm. The images were registered in remanence after application appropriate H_z_ magnetic field pulse. Domain images with higher resolution recorded for selected (indicated by yellow rectangulars numbered as 1-5 in Figs.S1a) Co thicknesses are presented in Fig.S1c. PMOKE hysteresis loops measured in regions distinguished by numbers 1-5 are presented in Fig.S1d.

Around SRT (*d*_Co_~1.48 nm) domain structure is below resolution of our PMOKE microscope. While decreasing *d*_Co_ below SRT one can observe an increase of black domain phase by “coercivity wall” [[[1]](#endnote-2)] propagation from SRT thickness region Fig.S1b,c. Higher resolution imaging Fig. S1c-4 shows the coercivity wall in the region 4 (Fig.S1b-4). Domain nucleation center density decreases while decreasing Co thickness (analyzing the image from right to left). Strong decrease nucleation center density (up to few on the area of 250x300μm^2^) is illustrated in Figs.S1c-1,2,3. So for the lower thickness magnetization reversal is dominated by domain wall propagation mechanism for higher domain wall nucleation. Similar effect is typical for magnetic film wedges see e.g. Au/Co/Au [[[2]](#endnote-3)], Au/Co/NiO [[[3]](#endnote-4)].


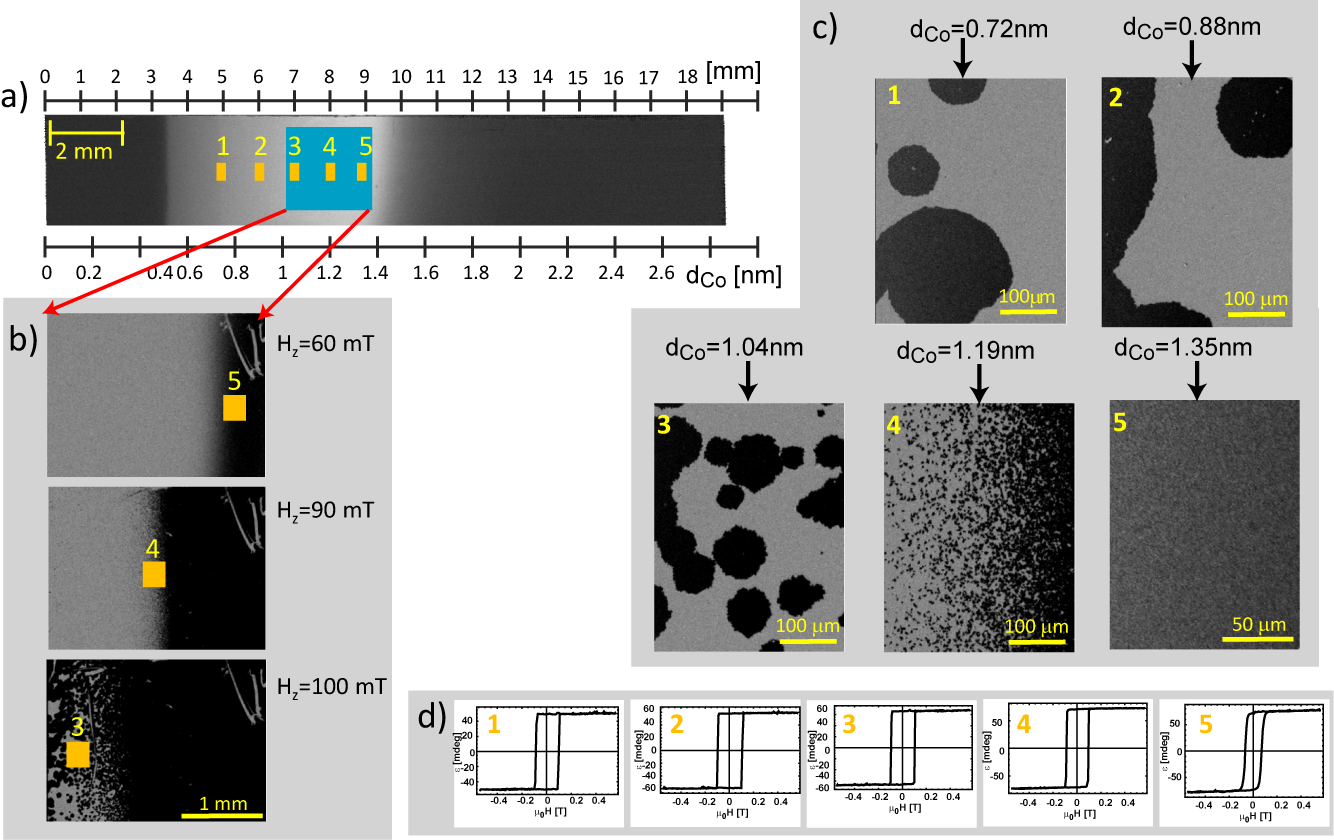


Figure S1. Magnetization reversal in NiO/Co(*d*_Co_)/Pt wedge with *d*_Co_ thickness below SRT: a) Differential PMOKE microscope images registered in remanence after saturation with an out-of-plane magnetic field similar as the inset to Fig.1 in the manuscript. Illustration of “black” domain area expansion registered: b) 2.8x2.0 mm^2^ area (low resolution) with central thickness *d*_Co_=1.2 nm, after selected magnetic field pulses H_Z_; c) 250x300 μm^2^ areas (high resolution). Region of interest are indicated by orange boxes in a) and b) and numbers 1-5. d) PMOKE hysteresis loops measured for selected (1-5) *d*_Co_ thicknesses.

***S2. XPS measurements***

50nm thick NiO deposited onto Si substrate in the same condition as for the full layer stack was studied by X-ray photoelectron spectroscopy (XPS). The sample was pumped down for 16 hours before the XPS measurements. X-ray photoelectron spectroscopy experiments were performed with a hemispherical analyzer SES R4000 (Gammadata Scienta, Uppsala, Sweden). The non-monochromatic Al Kα X-ray source (source energy: 1486.7 eV, electron gun: 12 kV, 15 mA) was used to generate core excitation. Photoelectrons were collected along the sample normal direction. The area of sample investigation was about 3 mm^2^. The survey scans were obtained at pass energy of 200 eV (with 250 meV step), whereas high-resolution spectra were gathered at pass energy of 100 eV (with 25 meV step). Under this condition, the energy resolution of the system was 0.9 eV, measured as a full width at half maximum (FWHM) for Ag 3d_5/2_ excitation line. All binding energy (BE) values were charge-corrected to the carbon C 1s excitation which was set at 285.0 eV.

Figure S2. X-ray photoelectron survey a) and b) Ni 2p and c) O 1s spectra for 50 nm NiO layer deposited by PLD.

Fig. S2a shows the XPS survey for the 50nm NiO layer deposited by PLD. The Ni 2p spectrum (Fig. S2b) is closely similar to those reported previously for high purity NiO in form of polycrystalline [[[4]](#endnote-5)]), freshly cleaved NiO (100) single crystal [[[5]](#endnote-6)], and for clean NiO after sputtering and reoxidation [[[6]](#endnote-7)]. It shows typical peaks assigned to the Ni^2+^ chemical state occurring together with typical shake-up satellites [[[7]](#endnote-8), Error: Reference source not found]. It should be emphasized that it is similar to that recorded for Au/Co/NiO system presented in our work [[[8]](#endnote-9)], where the NiO was deposited using the same procedure. Quantitative analysis was performed using the CasaXPS software. This analysis revealed a Ni(39%):O(39%):C(22%) composition. Therefore we may conclude that our procedure allows to deposit NiO layer with such composition. .

Note that the fabrication of perfect NiO stoichiometry 1:1 is difficult, therefore we cannot exclude the presence of Ni vacancies or surface absorbents (CO [Error: Reference source not found; [[9]](#endnote-10)], water[Error: Reference source not found]).

1. M. Kisielewski, A. Maziewski, M. Tekielak, A. Wawro, L. T. Baczewski, New Possibilities for Tuning Ultrathin Cobalt Film Magnetic Properties by a Noble Metal Overlayer, Phys. Rev. Let. 89 (2002) 087203, https://doi.org/10.1103/PhysRevLett.89.087203 [↑](#endnote-ref-2)
2. M. Kisielewski, Z. Kurant, M. Tekielak, W. Dobrogowski, A. Maziewski, A. Wawro, L.T. Baczewski, Magnetooptical micromagnetometry of ultrathin Co wedge in Au/Co/Au structures, Physica Status Solidi (a), vol.196, No.1 (2003) 129, https://doi.org/10.1002/pssa.200306370 [↑](#endnote-ref-3)
3. M. Kowacz, B. Anastaziak, M. Schmidt, F. Stobiecki, P. Kuświk, Strong Interfacial Perpendicular Magnetic Anisotropy in Exchange-Biased NiO/Co/Au and NiO/Co/NiO Layered Systems, Materials 14(5) (2021) 1237, https://doi.org/10.3390/ma14051237. [↑](#endnote-ref-4)
4. A.N. Mansour, Characterization of NiO by XPS, Surface Science Spectra 3 (1994) 231, https://doi.org/10.1116/1.1247751 [↑](#endnote-ref-5)
5. S. Uhlenbrockt, C. Scharfschwerdtt, M. Neumannt, G. Illing, H-J. Freund, The influence of defects on the Ni 2p and O 1s XPS of NiO, J. Phys.: Condens. Matter 4 (1992) 7973, https://doi.org/10.1088/0953-8984/4/40/009 [↑](#endnote-ref-6)
6. H.A.E Hagelin-Weaver, J.F Weaver, G. B Hoflund, G. N Salait, Electron energy loss spectroscopic investigation of Ni metal and NiO before and after surface reduction by Ar+ bombardment, Journal of Electron Spectroscopy and Related Phenomena 134 (2004) 139, https://doi.org/10.1016/j.elspec.2003.10.002 [↑](#endnote-ref-7)
7. S. Imaduddin, R. J. Lad, Epitaxial MgO(100) Film Grown on a NiO(100) Single Crystal Surface, Studied by XPS and UPS, Surface Science Spectra 4 (1996) 194, https://doi.org/10.1116/1.1247822 [↑](#endnote-ref-8)
8. P. Kuświk, M. Matczak, M. Kowacz, K. Szuba-Jabłoński, N. Michalak, B. Szymański, A. Ehresmann, F. Stobiecki, Asymmetric domain wall propagation caused by interfacial Dzyaloshinskii-Moriya interaction in exchange biased Au/Co/NiO layered system, Phys. Rev. B. 97 (2018) 024404, https://doi.org/10.1103/PhysRevB.97.024404. [↑](#endnote-ref-9)
9. S. Akhter, J.M. White, Stabilization Of C2Dx Fragments By Co On Ni(100), Surface Science 180 (1987) 19, https://doi.org/10.1016/0039-6028(87)90035-5 [↑](#endnote-ref-10)
